# Socio-economic predictors of undernutrition and anaemia in adolescent mothers in West and Central Africa

**DOI:** 10.7189/jogh.11.13007

**Published:** 2021-08-10

**Authors:** Vera Sagalova, Sebastian Vollmer, John Ntambi, Roger Sodjinou, Aline Simen-Kapeu, Till Bärnighausen, Noel Marie Zagre, Simeon Nanama

**Affiliations:** 1 Heidelberg Institute of Global Health, University of Heidelberg, Heidelberg, Germany; 2 Department of Economics and Centre for Modern Indian Studies, University of Göttingen, Göttingen, Germany; 3 UNICEF Area Representative for Gabon and São Tomé and Príncipe and to the ECCAS, Libreville, Gabon; 4 United Nations Children’s Fund (UNICEF) Gabon & São Tomé and Príncipe, Libreville, Gabon

## Abstract

**Background:**

Adolescence is a formative period when an individual acquires physical, cognitive, emotional, and social resources that are the foundation for later life, health, and well-being [1]. However, in West and Central African region, this trajectory is curtailed by early childbearing associated with an increased risk of undernutrition and anemia. Evidence on socio-economic determinants of anemia and undernutrition in adolescent mothers is limited. This paper aims to shed some light on this issue and, more specifically, assess the socio-economic determinants of anemia among childbearing adolescents in the region.

**Methods:**

For this observational study, we pooled data from all Demographic and Health Surveys (DHS) conducted in countries in West and Central Africa region between 1986 and 2017. Outcomes were undernutrition and anemia in adolescent mothers. Predictors were education, wealth, place of residence (rural/urban), and religion. Descriptive statistics were calculated using survey weights for individual surveys and in the pooled sample each country was additionally weighted with its population share. We estimated multiple regression models with and without primary sampling unit fixed effects for both outcomes. All regressions were linear probability models.

**Results:**

Having no formal education was the strongest predictor for both anemia and undernutrition. Belonging to the richest asset quintile was also associated with lower anemia and undernutrition prevalence in some specifications. While urban location of the mother was positively associated with anemia, there was no association with undernutrition.

**Conclusions:**

Overall, having any formal education emerged as a sole strong predictor of reduced adolescent maternal undernutrition and anemia. Promotion of female education can potentially serve as a high-impact intervention to improve adolescent girls’ health in the region. However, we cannot make conclusions about its causal impact based on this study alone.

Adolescence, the period between ages 10 and 19 years according to WHO’s (World Health Organization) definition [1] is a formative period when an individual acquires physical, cognitive, emotional and social resources that build the foundation for later life, health and well-being. It is also a critical period which defines trajectories into the next generation [2]. Yet every year, an estimated 11.7 million girls aged 15 to 19 years – an age group categorized as “older adolescents” – give birth worldwide [3], thus limiting their capabilities to expand the above-mentioned resources and changing their personal trajectory dramatically and indefinitely. UNICEF estimates adolescent birth rate in 2018 at 44 births per 1000 adolescents globally. This number masks tremendous regional differences. In West and Central Africa (WCA, UNICEF regional definition), the average adolescent birth rate is 115 live births per 1000 adolescent girls, with individual countries such as Chad and Central African Republic reaching rates of up to 179 or 229 births per 1000 adolescent girls respectively [4]. Birth rates for younger adolescents – 10 to 14 years – are not generally reported due to lack of reliable data. However, there are estimates that about 1 million births are incurred each year by girls below age 15 in low-resource settings, with the highest rates in Sub-Saharan Africa (SSA) [5].

The importance of monitoring and curbing early childbearing was recognized by the United Nations as indicated by the inclusion of adolescent birth rate in the Sustainable Development Goals indicators (SDG 3.7.2). Early childbearing has been shown to be a major driver of maternal and child mortality and to contribute to the perpetuation of the vicious cycle of ill-health and poverty [6]. The health risks to pregnant adolescent girls include reduced or ceased growth during pregnancy, weight loss, depleted fat body mass and lean body mass, iron deficiency (which is in turn linked with low birthweight), as well as pregnancy complications such as vesico-ureteric fistulae and obstructed labor. These risks are exacerbated if the girls are stunted prior to the pregnancy [1]. Health risks to adolescent mothers do not stop with birth but persist postpartum: adolescent mothers are shown to be at higher risks of postpartum infections and malaria and to show various signs of malnutrition (often in combination), such as iron-deficiency anemia, protein deficiency, or extreme weight loss [7].

Evidence on socio-economic determinants of anemia and undernutrition in adolescent mothers is limited and only available at national and sub-national levels. There is, however, a wide body of literature on this topic for the general population on a world- and country level [8,9] and on maternal anemia across all ages [10], as well as some evidence on adolescents in general without differentiating by pregnancy/maternity status [11-16]. Existing evidence at country level in LLMICS (Low and Lower Middle Income Countries) suggests that literacy may have a positive effect on nutritional status in the general population [17,18]. A study focused on the socio-economic determinants of nutritional status among married young women (<24 years) found that place of residence (rural vs urban), low wealth index score, low educational attainment, and early marriage increase the risk of being undernourished [19].

High prevalence of adolescent maternity in West and Central Africa coupled with increased risk of undernutrition and anemia in this region in general and in particular for young girls and new mothers highlight the need for further research in this area. This paper aims to shed some light on the issues above and, more specifically, assess the socio-economic determinants of anemia among childbearing adolescents in West and Central Africa region.

## METHODS

### Data sources

We pooled data from all Demographic and Health Surveys (DHS) conducted in West and Central African countries between 1986 and 2017. Descriptive statistics were calculated using only the most recent available survey per country; regressions were performed on all available data from all survey waves conducted past 1986. The pooled sample contains information on 43 122 adolescent mothers. However, biometric and anthropometric data are only available for a subset of surveys and subsets of eligible women within those surveys, Therefore, data on anemia and undernutrition (as measured by body mass index − BMI) was available for 9281 and 22 415 adolescent mothers respectively. The anemia sample covers the countries of Benin, Burkina Faso, Cameroon, Congo, Congo Democratic Republic, Cote d’Ivoire, Gabon, Gambia, Ghana, Guinea, Mali, Niger, São Tomé and Príncipe, Senegal, Sierra Leone, Togo while undernutrition sample contains data for the same set of countries with addition of Chad, Liberia, and Nigeria – so for 16 respectively 19 countries from overall 23 countries in the region.

BMI was calculated by dividing weight (in kilograms) by the square of height (in meters).

### Outcomes

Outcome variables are indicator variables for undernutrition and anemia. Undernutrition is defined by a BMI of less than 18.5 and because BMI is unreliable in pregnant women, we exclude those currently pregnant from undernutrition analyses (24% of adolescent mothers in the sample). Since we only look at young girls who already underwent pregnancy and childbirth, we deem the use of the same BMI cutoff point that is typically used to assess adult women’s nutritional status appropriate for them as well. Hemoglobin level was used to calculate an indicator variable for any form of anemia including mild, moderate, and severe. Hemoglobin level of less than 12g/dl for non-pregnant women and below 11g/dl for pregnant women denotes anemia in our study. These levels are already adjusted for altitude by DHS.

### Exposure

Exposure variables are education, wealth, place of residence (rural/urban) and religion. The education variable has the categories “none or less than primary”, “primary or incomplete secondary” and “secondary or higher”. In regression the category “none or less than primary” serves as reference category. Wealth is measured by asset index quintile categories: “poorest quintile”, “second quintile”, “middle quintile”, “fourth quintile”, and “richest quintile”. The poorest quintile serves as reference category in regressions. Rural/urban residence is expressed by a dummy variable for urban location, thus rural location being the reference group. In specifications where we control for primary sampling unit (PSU) fixed effects (FE), the location variable is dropped from the covariates as regional characteristics are already captured via the PSU. Religion is measured within the categories “Muslim”, “Christian”, and “none or other”. Muslim religion is used as reference category in regressions. While the authors do not think that religion per se is a driver for the outcomes of interest, it appears sensible that it might serve as a proxy for unobserved sociocultural practices, in particular with regards to meal preparation and intake (such as what kinds of meats are traditionally used, how are they prepared, is there a hierarchy in food intake or are all members of the family eating simultaneously – etc.) and hence this variable is included into some of the analyses.

### Statistical analysis

Descriptive statistics were calculated using survey weights for individual surveys and in the pooled sample each country was additionally weighted with its population share. All regressions were linear probability models. Some specifications include fixed effects at the primary sampling unit level to control for common characteristics of the local environment such as average living standards. In settings with large numbers of fixed effects, linear probability models are more robust than logit or probit models. All analyses as well as sample generation have been performed using Stata 14 statistical software package (StataCorp. 2015. Stata Statistical Software: Release 14. College Station, TX, USA).

## RESULTS

**Figure 1:**
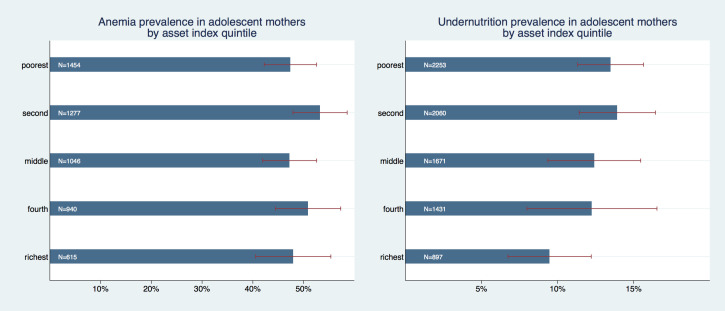
Prevalence of anemia and undernutrition in adolescent mothers by asset index quintile.

**Figure 1** summarizes the prevalence of anemia and undernutrition in adolescent mothers by asset index quintile. Graphically, no clear association emerges between asset wealth and anemia; the prevalence of anemia in the poorest asset index quintile was 48.3% (95% confidence interval (CI) = 43.6%-52.9%), in the richest quintile 48.1% (95% CI = 41.6%-54.9%) and all differences within subgroups were statistically insignificant (with *P*-values around 1).

The prevalence of undernutrition, on the other hand, revealed a strong negative association with asset wealth; the prevalence was highest in the two poorest quintiles: in the poorest quintile it was 13.5% (95% CI = 11.3%-15.6%) and slightly higher in the second quintile with 13.9% (95% CI = 11.4-16.4), whereas it was markedly lower with 9.5% (95% CI = 6.7%-12.2%) in the richest quintile. Within a pairwise mean comparison, the differences between the two highest asset wealth categories and the poorest were statistically significant (*P* = 0.001 for richest vs poorest and 0.012 for fourth vs poorest), as well as for richest vs second quintile *(P =* 0.007*).*

**Figure 2:**
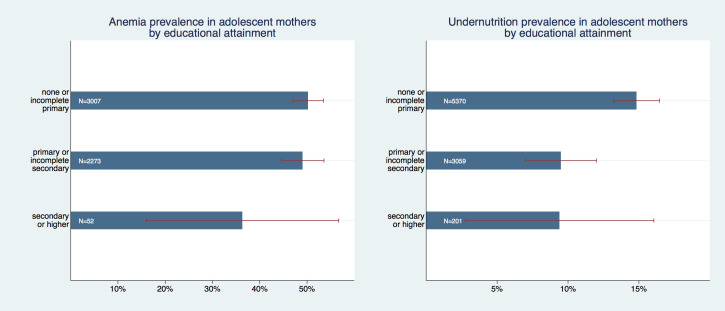
Prevalence of anemia and undernutrition in adolescent mothers by educational attainment.

**Figure 2** graphs the gradients of formal educational attainment. While we found differences in the prevalence of anemia for each educational subgroup, they were not statistically significant. The subgroup *“none or less than primary”* had an anemia prevalence of 50.2% (95% CI = 47%-53.5%), the subgroup *“primary or incomplete secondary” a* prevalence of 49% (95% CI = 44.5%-53.6%) and the subgroup *“secondary or higher”* a prevalence of 36.3% (95% CI = 16%-56.7%). However, when running mean comparisons on a more detailed education variable with six categories – none, incomplete primary, complete primary, incomplete secondary, complete secondary, and higher education – the difference between the highest and all but second highest category was statistically significant with *P* < 0.001.

The difference in undernutrition was statistically significant between the reference group *(“none or less than primary”)* and the other two subgroups (*P* < 0.001 in both pairs). The reference group had an anemia prevalence of 14.8% (95% CI = 13.2%-16.5%), while the subgroup *“primary or incomplete secondary”* had a prevalence of 9.5% (95% CI = 7%-12%), and “secondary or higher” had a very similar point estimate of 9.4%, however a much larger confidence interval (95% CI = 2.8%-16.1%). Notably, the number of observations in the highest educational category was extremely low (in relative and absolute terms) with only 52 observations or below 1% in the anemia analysis and 201 observations or 2.3% in the undernutrition analysis.

**Figure 3:**
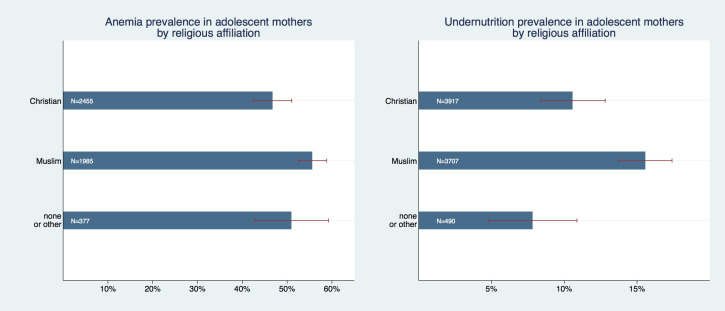
Prevalence of anemia and undernutrition in adolescent mothers by religious affiliation.

We found the prevalence of anemia and undernutrition to differ across various (large) religious groups; **Figure 3** shows that Muslims had a higher prevalence of both anemia (55.8%, 95% CI = 52.7%-58.9%) and undernutrition (15.6%, 95% CI = 13.7%-20.5%) as compared to Christians (47.1%, 95% CI = 43.2%-51%; and 10.8%, 95% CI = 8.4%-12.8%, respectively) or women with traditional, unknown, or no religion. Differences in undernutrition prevalence between the Muslim and the other two groups were statistically significant (*P* < 0.002) but all pairwise group comparisons were insignificant at 95% level for anemia.

**Figure 4:**
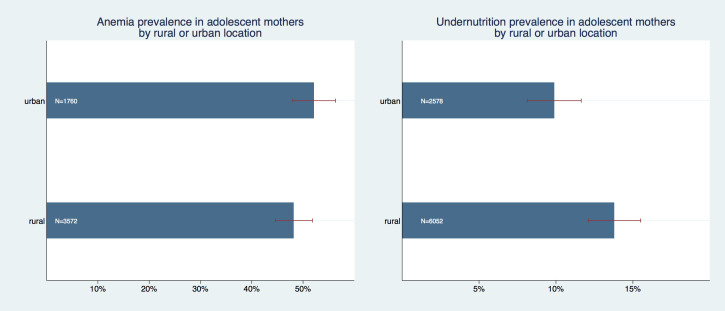
Prevalence of anemia and undernutrition in adolescent mothers by location.

Place of maternal residence made a statistically significant difference for undernutrition (*P* < 0.001) but not for anemia (*P* = 0.690) (**Figure 4**). Adolescent mothers from rural locations had a lower anemia prevalence of 48.1% (95% CI = 44.6%-51.8%) compared with urban adolescent mothers’ prevalence of 52.1% (95% CI = 47.9%-56.3%). Undernutrition prevalence was on the contrary lower for urban mothers with 10.3% (95% CI = 8.1%-11.6%) but higher for rural mother with 13.8% (95% CI = 12.1%-15.5%).

Some of these graphical patterns were confirmed in a multiple regression analysis in which all factors were simultaneously controlled for (c.f. specifications (1) and (3) in **Table 1**). Having any formal education (vs having no education at all) was associated with a 4-percentage point reduction in anemia probability and a 5-percentage point reduction in undernutrition probability (*P* < 0.001). Belonging to the richest asset quintile was associated with a 7-percentage points lower anemia probability *(P <* 0.001). Urban location of the mother was associated with an increased anemia probability of 4.7 percentage points (*P* = 0.001) but it was not found to be a statistically significant predictor of undernutrition. The coefficients only marginally changed when the models were adjusted for primary sampling unit fixed effects, which control for common characteristics of the local environment (c.f. specifications (2) and (4) in **Table 1**). In these fixed effects models, having any formal education was associated with a 3.1 percentage points decrease in anemia and 4.8 percentage points decrease in undernutrition probability *(P <* 0.001*).*

**Table 1:** Multiple regression analysis of anemia and undernutrition on select socio-economic indicators*

	Anemia		Undernutrition	
Educational attainment:				
Any formal education	-0.0401§ (0.011)	-0.0314‡ (0.012)	-0.0530§ (0.005)	-0.0481§ (0.006)
Wealth quintile Reference: lowest quintile):				
Second quintile	0.0118 (0.015)	0.0149 (0.016)	0.00250 (0.008)	-0.000934 (0.009)
Middle quintile	0.000657 (0.016)	0.00153 (0.017)	-0.00101 (0.008)	-0.000730 (0.009)
Fourth quintile	-0.0451† (0.018)	-0.0331 (0.018)	-0.0159 (0.009)	-0.0187 (0.010)
Richest quintile	-0.0699§ (0.021)	-0.0344 (0.021)	-0.0148 (0.011)	-0.0230† (0.010)
Location of residence (reference: rural):				
Urban	0.0465‡ (0.014)		-0.00154 (0.007)	
Constant	0.574§ (0.011)	0.579§ (0.010)	0.152§ (0.007)	0.152§ (0.006)
PSU FE	No	Yes	No	Yes
Observations	8882	8882	15 562	15 562

PSU FE – primary sampling unit fixed effects

*Standard errors in parentheses.

†*P* < 0.05.

‡*P* < 0.01.

§*P* < 0.001

**Figure 5:**
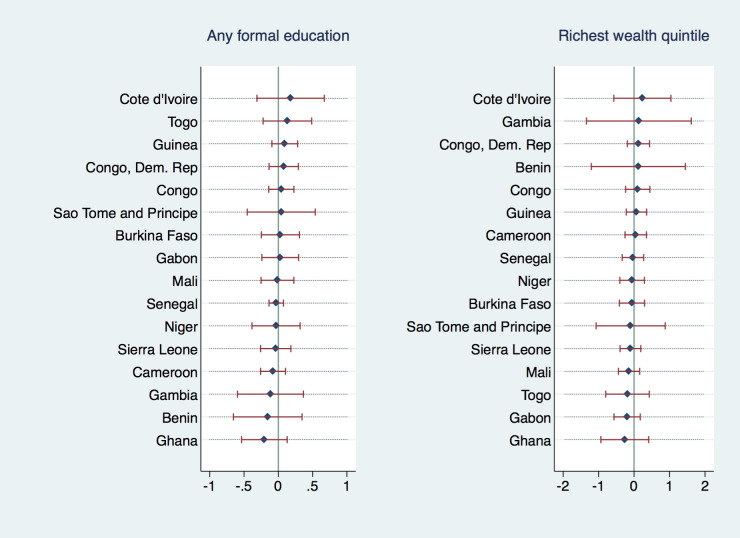
Coefficients from adjusted model with PSU FE, by country, outcome variable: anemia in adolescent mothers

**Figure 6:**
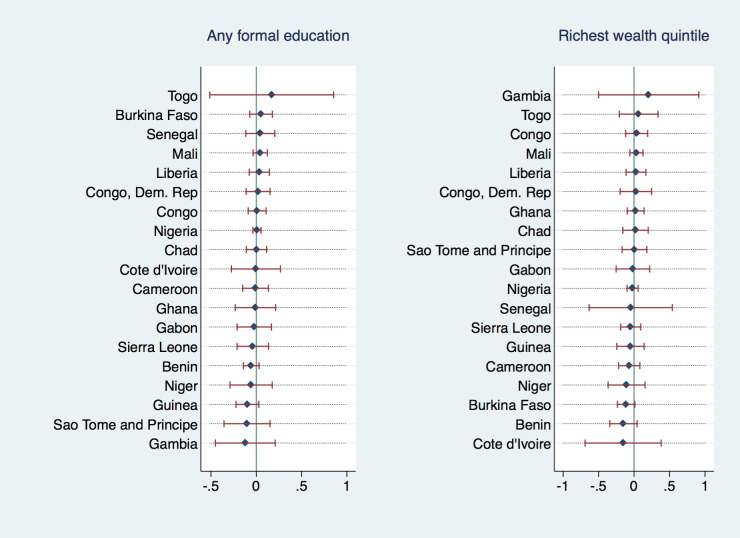
Coefficients from adjusted model with PSU FE, by country, outcome variable: undernutrition in adolescent mothers.

**Figure 5** and **Figure 6** explore the heterogeneity in the association of wealth and education with the outcome variables across countries. They depict individual country-level coefficients for binary indicators *“having any formal education”* and *“belonging to the richest wealth quintile”* from multiple regression analyses of undernutrition and anemia (analogous to **Table 1**, Column (2) and (4)). While we did not find any of the individual country coefficients to be significant, there was heterogeneity in the direction and magnitude of the coefficients; Cote d’Ivoire displayed the highest positive association between anemia prevalence and formal education or asset wealth, implying that formal education was actually correlated with an increased probability of anemia and undernutrition in this country. Most coefficients, however, were very close to zero or negative with Ghana displaying the strongest negative correlation between anemia and education or wealth. In the undernutrition regressions, most individual coefficients have been, again, negative or very close to zero, with Togo/Gambia being notable examples with a positive (yet insignificant) association between formal education/wealth and undernutrition.

## DISCUSSION

Our regression analyses revealed the socio-economic predictor ‘Any formal education’ to have significant negative association with both outcomes throughout both model specifications (with and without PSU FE) used. The fact that ‘any formal education’, which is defined as educational attainment of at least completed primary education, is sufficient to establish this correlation suggests that a crucial threshold is reached somewhere early in the educational process. ‘Household wealth’ had a significant association with both outcomes in some of the specifications. Within a smaller sample, containing only the most recent survey per country, we found gradients in undernutrition along asset wealth- and educational variables, where higher-ranked population groups were consistently and statistically significantly better off in terms of their nutritional status. However, we could not establish any statistically meaningful gradients in anemia; only when using a more detailed variable for educational attainment (results not presented) such a gradient could be observed with the highest educational category (higher education) being statistically significantly different from all other, lower categories but the second-highest (complete secondary). The fact that we were not able to establish any apparent association between anemia and household wealth appears somewhat puzzling – such a linkage has been established for instance for women of reproductive age using DHS data for South- and Southeast Asian countries [20]. It appears intuitive that some of the most prominent causes of anemia in low-income countries such as inadequate nutritional iron or folate intake (eg, caused by low or no meat consumption), intestinal parasite pressure, and exposure to malaria [21] will be alleviated with certain lifestyle changes pertinent to an increase in disposable income. This provides an interesting starting point for further investigation. Moreover, while the usage of religion as a predictor for health outcomes is not intuitively straight-forward and requires further justification, which is beyond the scope of this work, we found statistically significant and quite pronounced differences between Christian and Muslim adolescent mothers in both outcomes. Variables for religious affiliation, though generally available in large household surveys, appear to rarely be utilized in analyses of nutritional health outcomes. We were unable to identify any multi-country studies focusing on undernutrition or anemia in adults or adolescents in low-resource settings that reported any findings along religious affiliation lines. Our own previous work with large cross-sectional data sets in WCA suggests that there is at least some association between household religion and nutrition-specific practices and that, on average, Christian population in the region has more favorable outcomes than Muslim population, even after controlling for education, wealth, and local characteristics [22].

Our findings suggest that in order to better understand the driving forces behind anemia and undernutrition in young mothers, further evidence on socio-cultural practices that affect nutrition would be highly beneficial in this culturally diverse, heterogeneous region.

While the pooled sample is large enough to find statistically significant associations, when looking at 19 (16 in anemia) countries in the region individually, their respective sample sizes are relatively small, limiting the statistical power of the country level regressions. It is thus not possible to draw conclusions about the heterogeneity of coefficients within our targeted region on a disaggregated level, as none of the coefficients of interest is statistically significant in individual country regressions. In addition, while we generally refer to ‘adolescents’ throughout the present text, it is important to stress that existing evidence only focuses on older adolescent girls aged 15-19, while systematic anthropometric and biomarker information on boys is lacking entirely and most surveys also do not cover younger adolescent girls aged 10-14.

This study is based on pooled data from cross-sectional surveys and does not allow making any causal inferences. Moreover, pregnant adolescent girls, despite being a group of interest for this type of analysis, were excluded from analyses on undernutrition for methodological reasons (lack of appropriate BMI-cutoffs). Aforementioned limitations notwithstanding, these results provide useful insights into some of the factors underlying anemia and undernutrition at the sub-regional and country level in West and Central Africa. Furthermore, the present study highlights the need for further research on the determinants of the differential prevalence rates among adolescent mothers.

## CONCLUSION

Overall, within our analyses, only having any formal education emerged as a strong correlate of both adolescent maternal undernutrition and anemia. While we do not make any judgment about causation, plethora of pathways are imaginable as to how education serves as a protective factor against these adverse health outcomes and lack thereof, in contrast, is associated with higher prevalence rates. Coupled with the finding from our previous work that schooling is also a protective factor against adolescent pregnancy [23], increasing efforts put into promotion of female education appears to be a worthwhile cause per se and based on these findings could potentially serve as a high-impact intervention to improve adolescent girls’ health in low-resource settings. Moreover, to understand the pathways between religious and cultural practices and nutritional outcomes in adolescent mothers, novel research tools and in particular appropriate questionnaires addressing these issues need to be developed and administered within large household surveys. Lastly, large household surveys in their current state barely allow moving beyond descriptive research on adolescent maternal health and nutrition on country level in West and Central African region. Meaningful inferences on these issues would require larger sample sizes within this subpopulation and a specific targeting of adolescent girls as part of the sampling strategy.
